# Assessment of Symptoms of Grapevine Leafroll Disease and Relationship with Yield and Quality of Pinot Noir Grape Must in a 10-Year Study Period

**DOI:** 10.3390/plants12112127

**Published:** 2023-05-27

**Authors:** Cristina Cabaleiro, Ana M. Pesqueira, Julián J. García-Berrios

**Affiliations:** Escuela Politécnica Superior de Ingeniería, Departamento de Producción Vegetal y Proyectos de Ingeniería, Campus Terra, Universidade de Santiago de Compostela, 27002 Lugo, Spain

**Keywords:** grapevine, virus, GLRaV, symptoms, severity, incidence, losses, leafroll

## Abstract

Grapevine leafroll disease (GLD) is caused by one or more of the Grapevine leafroll-associated viruses (GLRaVs). GLD’s symptoms are expected to be evident in indicator cultivars, regardless of the GLRaV(s) involved. In the present study, disease incidence (I) and severity (S), symptoms before veraison (Sy < V), a disease severity index (DSI) and an earliness index (EI) (2013–2022) were recorded in order to examine the factors affecting the evolution of GLD in Pinot noir graft inoculated with scions infected with GLRaV-3 that, in origin, showed a diversity of GLD symptoms. Strong correlations between I and S (r = 0.94) and between Sy < V and EI (r = 0.94) were observed; early symptoms proved good predictors of incidence and severity after veraison and of yield and sugar content of the must. The environmental conditions and time after infection did not modify the wide range of symptoms (I: 0–81.5%; S: 0.1–4) that corresponded with the variation in losses (<0–88% for yield and <0–24% for sugar content). With all other factors being constant, the significant differences between plants were mainly due to the GLRaVs present. Plants infected with some GLRaV-3 isolates always had mild symptoms or remained asymptomatic 10 years after grafting but remained a source of infection for GLRaV vectors.

## 1. Introduction

Grapevine leafroll disease (GLD) is a complex disease with several associated virus species, most of which are ampeloviruses. Grapevine leafroll-associated virus-3 (GLRaV-3) is the best known of these viruses, the most widespread and the most frequently associated with GLD symptoms and damage, along with GLRaV-1 [1,2]. Mixed infections of several GLRaV are very common [3]. Unlike GLRaV-1 and GLRaV-3, GLRaV-2, -4 and -7 are often associated with asymptomatic plants or with mild GLD symptoms with apparently little impact on either fruit yield or quality [4]. When GLRaV-3 occurs along with one or more of the other leafroll viruses, the GLD symptoms and the impact on yield and quality can vary. Recent studies involving dsRNA sequencing of symptomatic and asymptomatic grapevine leaves of different cultivars revealed a very variable virome (viruses and viroids) in all plants [5]. The presence of other diseases may also be an important factor. In a review paper published in 2021 [4] concluded that when investigating the effects of a specific virus on the grapevine host, the pathobiome of the affected plant, i.e., the population of co-existing viruses and viral strains in the infected grapevine, should also be considered.

As with many other viral diseases, the expression of the GLD symptoms has been associated with different factors, many of which are not related to the virus itself but to the location, environmental conditions, age, canopy management, cultivar and rootstock [2,6,7,8,9,10,11,12]. In a cool climate in New Zealand, leafroll symptoms could not be used as the only justification for roguing GLD infected plants in young vineyards with Sauvignon blanc and Pinot gris vine plants, but it was possible to do so with Merlot and Pinot noir vine plants [13]. There are also reports of mild symptoms not leading to damage in some cultivars; for example, in Australia a mild strain of GLRaV-3 was detected in desirable clones of Crimson Seedless table grapes [14]; one of the isolates studied by [15] did not show any leafroll symptoms in the vineyard of origin.

Several grapevine cultivars are assumed to display unmistakable symptoms of leafroll disease: Cabernet Franc, Cabernet Sauvignon, Pinot noir, Merlot, Barbera, Gamay, and hybrids such as LN33 and others [16]. As a result, biological indexing of selected clones is done by grafting the clones onto any of those “indicators”, which will show symptoms independently of the leafroll virus present, although the symptoms may not appear for several years [6,17]. A positive serological or molecular test does not definitely indicate how the disease will develop; this can be done by biological indexing, which is considered a very sensitive method for the detection and bio-amplification of viruses [17]. However, there are some reports of asymptomatic indicators after graft transmission of some GLRaVs, mainly GLRaV-2 and GLRaV-7 [18]. In GLRaV-3, indexing is highly reliable but there are some reports of infected indicators with only mild symptoms. In a pot assay conducted in the Balearic Islands, some infected Cabernet sauvignon vine plants were asymptomatic [19]; a variant found in Idaho (USA), named ID45, did not cause any foliar symptoms in Cabernet Sauvignon in autumn [20]. In a long-term study, Pinot noir showed mild or strong symptoms when infected with GLRaV-1 or a combination of both GLRaV-1 and 3 [11]; in the same study, the authors confirmed the value of High-Throughput Sequencing (HTS), which is faster and more sensitive than biological indexing; however, as already mentioned, virus detection does not necessarily equate with disease expresion or evolution [21]. In plants propagated vegetatively for thousands of years and grafted on species and hybrids of different origin, multiple combinations of viruses, viroids and other systemic pathogens are expected; interpretation of the possible effects in relation to GLD requires a good understanding of the relationship between symptoms and damage, for which long term assays are essential. In a study comparing GLD caused by infection with different individual and mixed GLRaVs [22], we realized that collecting detailed information about the development of symptoms and disease evolution in a population with known high variability would help to determine how much of the variability is explained by the viruses themselves.

The corresponding research began in 2006, when we carried out a survey of several wine grape appellations in Galicia (NW Spain) searching for variability in GLRaV-3 in local red cultivars. We investigated symptom expression at veraison and harvest and the effect on yield and quality during several years, obtaining very variable results [7]. For the present 10-year study, we selected 10 plants from those heterogeneous plants, to represent as wide as possible a range of leafroll symptoms according to the data recorded at the original location; all the plants were infected with at least one strain of the GLRaV-3 virus and the molecular variability of the different strains was examined, as previously reported [15]. The plants showed strong, mild or no GLD symptoms in the original location, and symptoms appeared earlier or later in the season. By grafting the plant onto the same clone of the Pinot noir indicator at the same location, we aimed to assess the evolution of GLD symptom expression during a long enough period to determine whether specific symptoms were related to the year of infection, annual environmental conditions and/or the productive response of the vines.

## 2. Results

### 2.1. Weather Conditions

The meteorological data place the vineyard in the 1b region/class, according to the Winkler index (1111–1389 °C). For most years, the HI index places the vineyard in the “temperate” (HI-1) class, although some years were “cool” (HI−2) and some “temperate warm” (HI+1). In 2017, spring frost destroyed the canopy, affecting all data collected by delaying the onset of symptoms and halting grape production (Table 1). In addition to the annual data shown in Table 1, the temperatures and rainfall for the months and years when observations of symptoms were carried out are shown in Table 2.

### 2.2. Evaluation of GLD Symptoms and Interpretation

**Figure 1:**
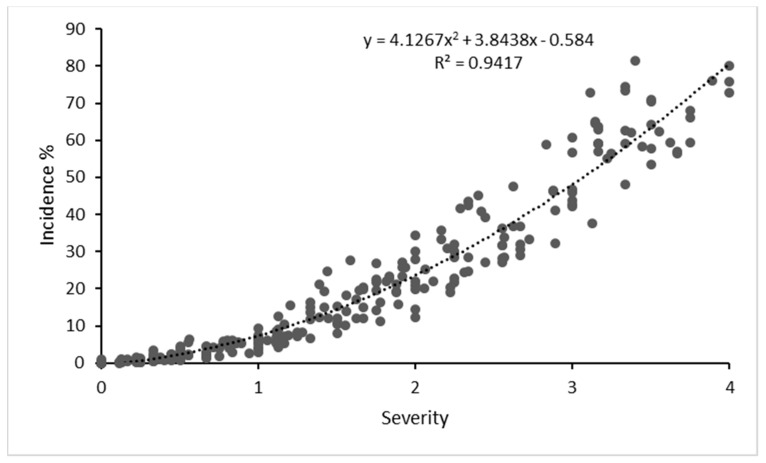
Scatter plot of disease severity vs. disease incidence: mean data for June, July, August and September (2013−2022) for all infected plants.

Disease severity and incidence were quite well correlated: in plants with stronger symptoms, a larger area of the canopy was affected (Figure 1). Pearson’s correlation coefficient was positive and statistically significant (r > 0.94; *p* < 0.01; *n* = 277). A polynomic curve fitted the data better than a linear curve (R^2^ = 0.94), because it is rare for 100% of the canopy to display leafroll symptoms, even in the most severely affected plants.

**Figure 2:**
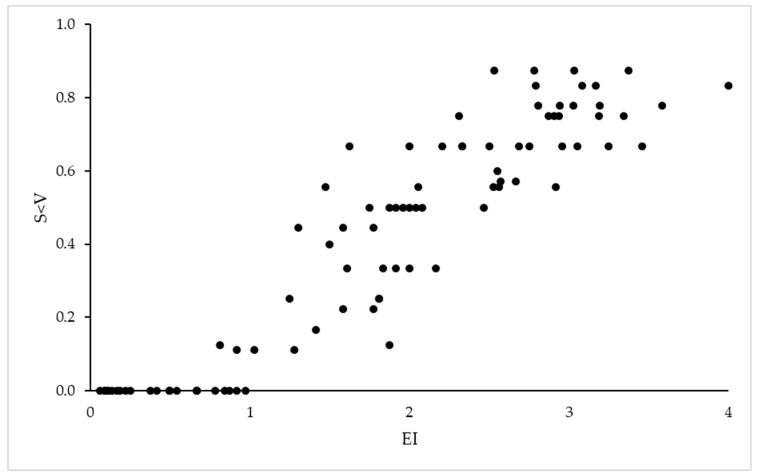
Scatter plot of the mean number of plants with GLD symptoms before veraison (Sy < V) versus the mean earliness index (EI) for the same plants (2014–2022).

**Figure 3:**
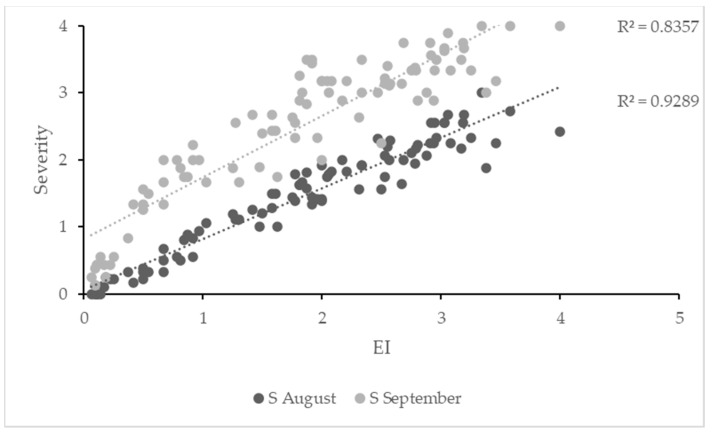
Scatterplot of EI vs. Severity in August and September (2013–2022) and linear regression lines.

The correlation between the two indices of early symptom expression was positive (r = 0.94; *p* < 0.01) (Figure 2). As EI was calculated with S values for the four observation dates, it yielded a better correlation with the global I*S index (r = 0.88) than Sy < V (r = 0.77), although both were highly significant (*p* < 0.001); the same occurred when the analysis was carried out with S and I separately. Generally, earlier symptoms (higher Sy < V and higher EI) led to more and stronger symptoms after veraison, and plants with no symptoms in June or with low severity in July had no symptoms or only mild ones at harvest. The EI-S correlation between July and September was highly significant (r > 0.90); however, the highest correlation (Figure 3) was between EI and S in mid-August (r = 0.96) when plants were in full veraison. The same correlations for Sy < V were more dispersed but still significant. With EI as a fixed factor, the regressions with S in August and September as dependent variables were significant (R^2^ = 0.92, SEM = 0.22; *p* < 0.001 for August; R^2^ = 0.84, SEM = 0.64; *p* < 0.001 for September).

**Figure 4:**
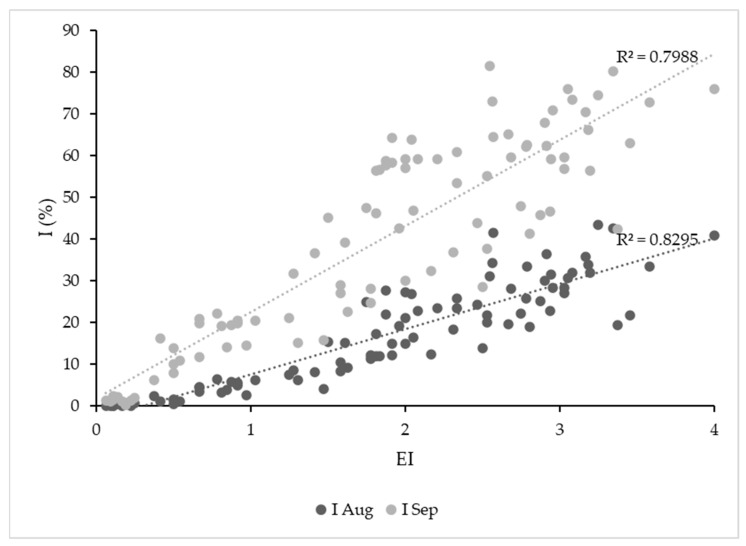
Scatter plot of Earliness Index (EI) versus disease incidence (I) in August and September (2014–2022) and linear regression lines.

Both earliness indices were strongly correlated with I at the end of the summer (r = 0.91 for August and r = 0.89 in September) (Figure 4).

### 2.3. Symptoms and Environmental Conditions

The environmental factor that most strongly affected the appearance, incidence and severity of sympoms was the spring frost in 2017, with abnormally low values for June, July and August due to destruction of the vines in April and a general delay in the development of the canopy, with no grapes produced; plants had recovered by September and displayed the typical post-veraison symptoms.

**Figure 5:**
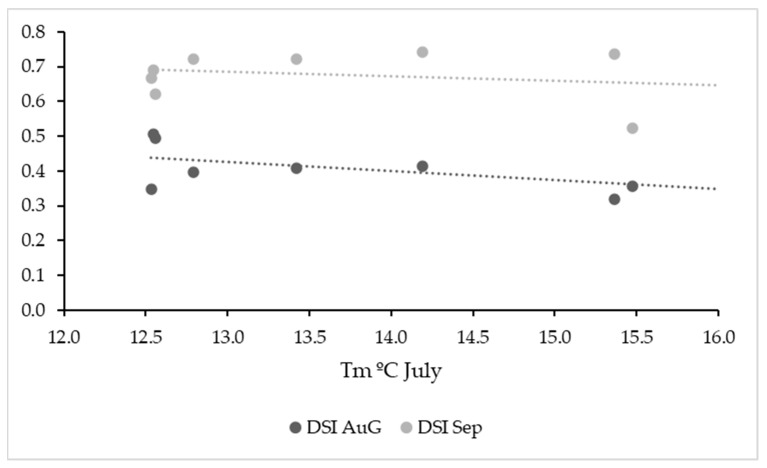
Scatter plots of average Tm in July (2014–2022) and mean DSI in observations in August and September. Trend lines are also shown.

**Figure 6:**
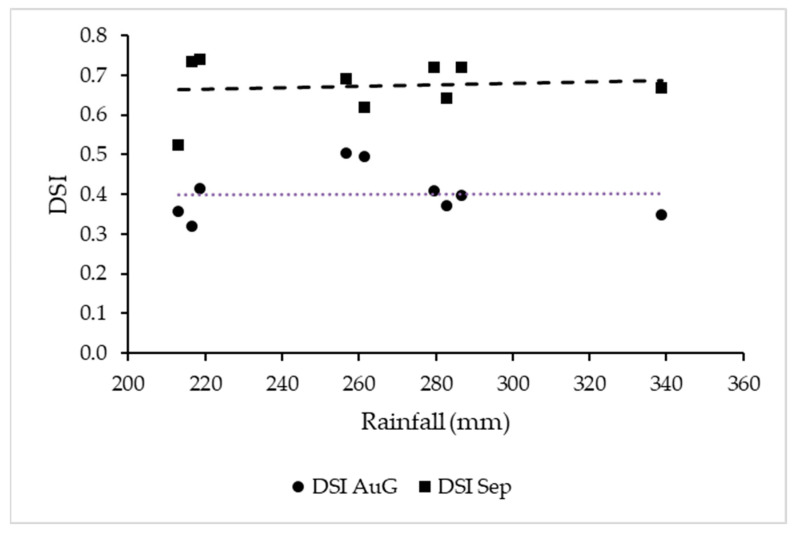
Scatter plots of the rainfall (mm) between April and September 2014–2022 vs. mean DSI in observations in August and September. Trend lines are also shown.

Temperature was not a determining factor considering the whole period; the correlation between Winkler´s and the mean DSI of each year was positive but not significant (r = 0.23; *p* > 0.05). For symptoms in September, the correlation was negative and not significant (r = −0.39; *p* < 0.3). There were no clear correlations between temperatures and no consistent trends for each month or the month before the observation and the DSI (Figure 5), and there was also no clear relationship between temperatures in June or July and earlier symptom expression throughout the 10-year study period. Rainfall during the vegetative period was also not a determining factor for the DSI after veraison (Figure 6).

### 2.4. Symptoms/Damages and Years after Graft-Inoculation

**Figure 7:**
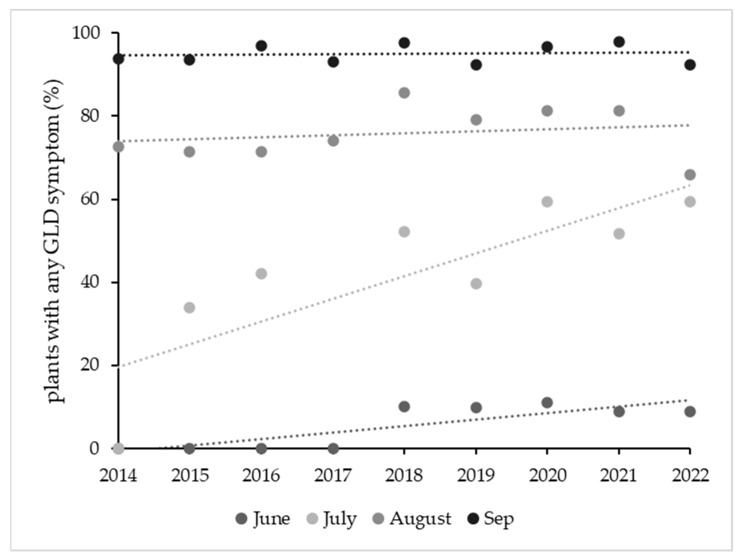
Percentage of plants with GLD symptoms (presence/absence) in June, July, August and September between 2014 and 2022.

**Figure 8:**
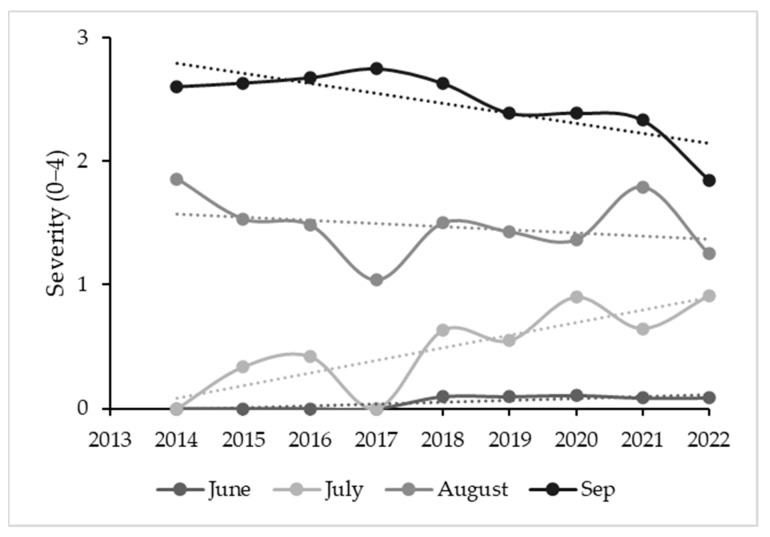
Change in severity of symptoms between 2014 and 2022. Mean values for positive plants on any observation date. Trend lines are shown for each month of observation.

**Figure 9:**
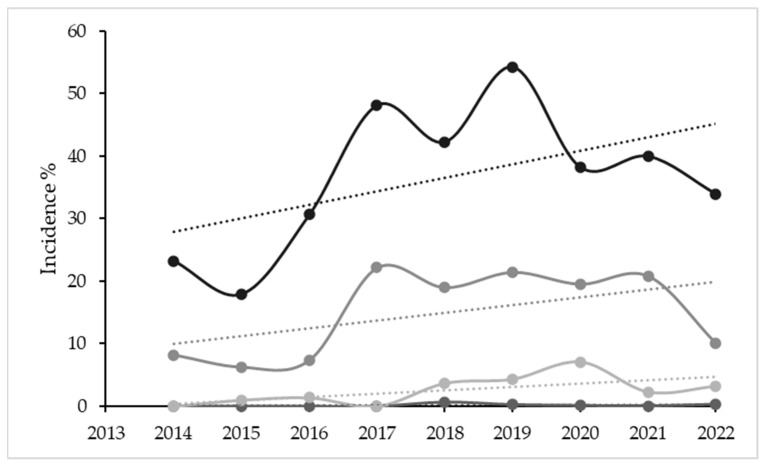
Change in incidence of GLD symptoms between 2014 and 2022. Mean values for date of observation and trend lines are shown.

Over time, the percentage of plants displaying any type of GLD symptoms (ranging from mild to strong) after veraison was similar in August and September. In June, there was a significant increase in this percentage (F = 15.2; *p* < 0.01) because some plants started to consistently show early symptoms since 2018. In July, the percentage of plants with symptoms increased significantly between 2014 and 2022 (Figure 7). The disease severity index for June and especially July increased between 2014 and 2022 (with anomalous data for 2017). There were no significant changes in August throughout the years, but there were two clear anomalies, one in 2017 and another in August 2021, with no clear explanation for the most severe symptoms. The tendency for S to have lower values in September throughout the study period was significant (r = 0.84; *p* < 0.005) (Figure 8). Correlations for disease incidence were not significant and followed a positive, but irregular trend (Figure 9).

**Figure 10:**
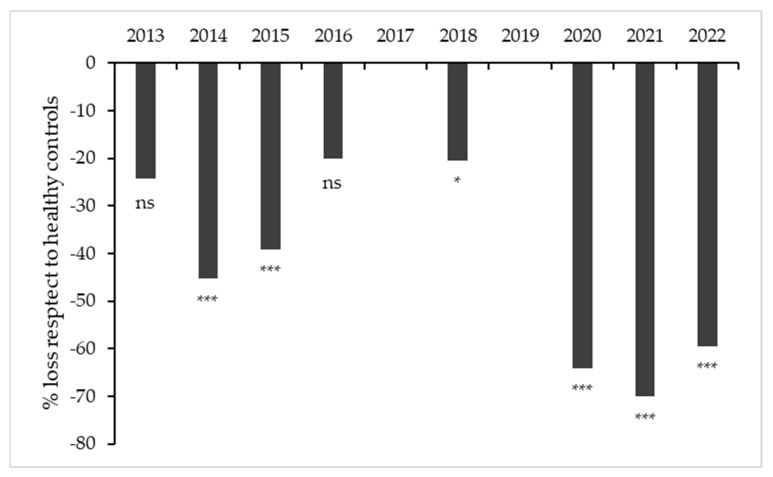
Change in the mean percentage of yield lost relative to the yield of the healthy controls between 2013 and 2022; comparisons for yield of infected vs. healthy plants; ns, non-significant; * significant with *p* < 0.05; *** significant with *p* < 0.001.

**Figure 11:**
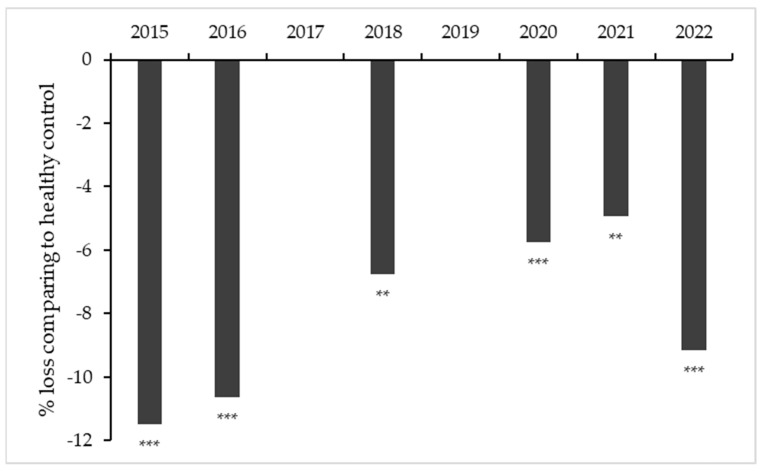
Change in the percentage decrease in sugar content (°Brix) of the must relative to the values for the leafroll free controls for the available harvests (2015, 2016, 2018, 2020–2022). comparisons for infected vs. healthy plants; ns, non-significant; ** significant with *p* < 0.01; *** significant with *p* < 0.001.

The plant yield and quality of the must for the 2013–2022 harvests was as variable as expected in a non-irrigated vineyard under variable environmental conditions. The mean yield from leafroll free plants was 1.6 kg/plant (sd ± 0.3) and mean yield infected plants, 0.9 kg/plant (sd ± 0.4); the differences in yield relative to the healthy controls ranged from −13% to 88%. The percentage of harvest loss relative to the healthy control plants varied for each harvest and reached very high levels in the last three years (Figure 10). A similar trend was observed in the number of clusters (). The mean decrease in sugar content in infected vs negative control plants since 2015 is shown in Figure 11.

### 2.5. Symptom Indices and Plant Performance

**Figure 12:**
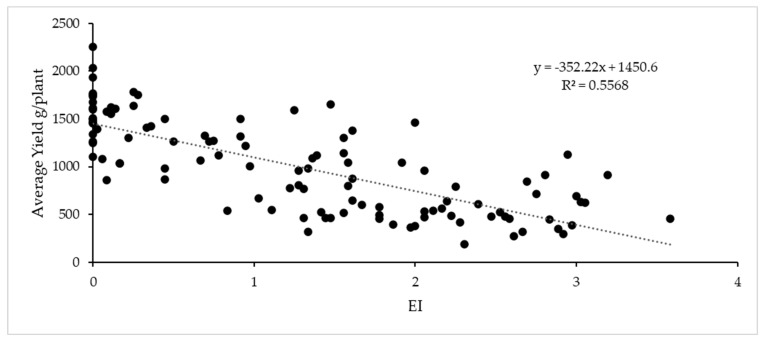
Scatterplot and linear regression line for data on yield (average g/plant for harvest 2013–2022) and the average index of earliness (EI). R^2^ = 0.56; SEM = 322.1; F = 134 *p* < 0.001.

**Figure 13:**
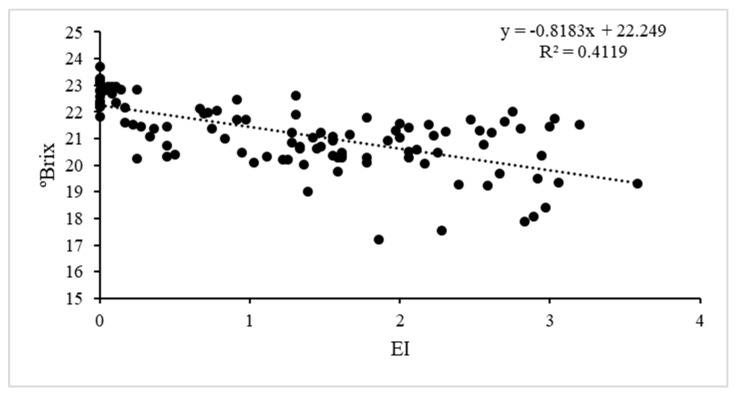
Scatterplot and regression line for sugar content of the must (°Brix) and the mean earliness index (EI) values. R^2^ = 0.41, SEM = 1.01; F = 75 *p* < 0.001.

The indices of symptom precocity and disease incidence and severity on different observation dates were significantly and negatively correlated with the mean yield of plants and sugar content (Table 3). Earlier symptoms were closely correlated with poorer plant performance (Figure 12 and Figure 13) and the same applied to severity and spread of symptoms. The total acidity was not correlated with early symptoms, but was correlated with symptoms in August and September, and r was always positive: the must from plants with GLD symptoms was more acidic. The product kg*°Brix closely correlated with all indices (Table 3).

The Pearson’s correlation coefficients shown in Table 3 were calculated with the mean values for all years, but when calculated with the symptoms and harvest data for each year, the results were always significant.

**Figure 14:**
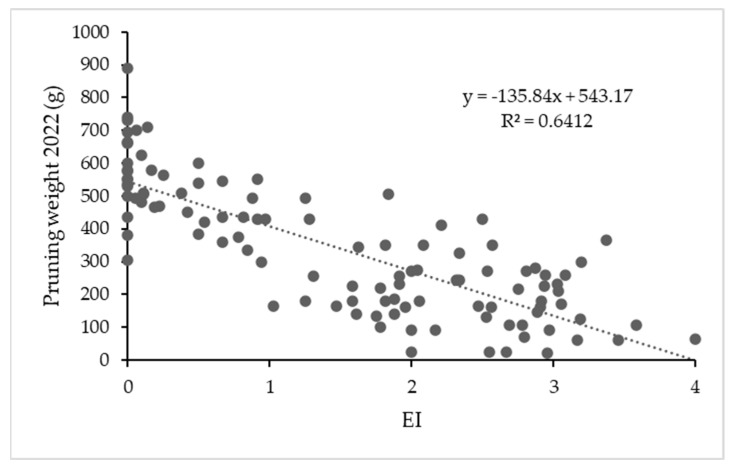
Scatterplot with mean earliness index (EI) values for all plants and years vs. pruning weight after the last harvest (2022/23) and linear regression line.

Ten years after grafting, plants that as an average had earlier, more severe and more widespread symptoms are declining and dying, and pruning weight in some of them was close to 0 for the most affected plants. In Figure 14, the graph of pruning weight vs. EI is shown.

### 2.6. Symptoms and Damage according to Virus Isolate

The determining factor of the variability of symptoms observed and of the quantified damage in harvest year after year, is the presence of GLRaV-3 of different origins and their combinations with GLRaV-1 and/or 2. The statistical analysis (ANOVA) for all the measured parameters was very significant when the fix factor was the virus isolate (Table 4 and Table 5).

## 3. Discussion

Grapevine virus diseases are generally only of concern for growers when very striking symptoms appear in the field, especially those affecting grape clusters (millerandage), as observed with GFLV and other nepoviruses. Although information has been compiled in the last decades regarding the economic impact of GLD [10,23,24,25,26,27] winegrowers’ concern about GLD does not seem to have increased greatly in traditional vineyards in Europe [28]. Winegrowers do not readily associate loss of yield or quality with GLD symptoms, to the point, that in some cases, leaf rolling and reddening or yellowing are considered characteristic of cultivars and to be a natural process occurring in vineyards in autumn. This is because GLD generally has an asymptomatic period followed by a symptomatic phase that usually occurs post-veraison due to different metabolic processes [4]; as clusters change colour at veraison, it seems only “natural” that the canopy will do the same. This process is common in most red cultivars but not in most white ones [13].

By using several indices to assess the GLD, we confirmed a significant relationship between disease incidence and severity; thus, as symptoms spread in the canopy, they become more severe in the affected leaves. The relationship between symptoms and damage was also significant; late and mild or no symptoms did not affect the plant yield after 10 years, and early and strong symptoms affected plant development and hampered plant production and survival after 10 years, which is a very short period relative to the expected lifetime of a vineyard. Roguing is therefore fully justified in the case of early and strong symptoms and must be performed as early as possible, since our data indicate that such symptoms predict economic losses. If replanting is necessary, it is better to do it during the first years after planting [29].

Among the indices used, all were closely related to damages, but the most accurate and practical was usually the earlier or later appearance of symptoms. Mild symptoms at harvest start late in the season and severe symptoms much earlier, and almost always before veraison; although the earliness index (EI) usually more strongly correlated with symptoms than Sy < V, the second is a simpler way to predict damage during the season. In [11], symptoms are reported in the most aggressive isolates up to 6 weeks before veraison, but in the present study, some plants showed symptoms as early as mid-June, i.e., 7–8 weeks before veraison.

As an indicator plant, Pinot noir was expected to give a more uniform response regarding the onset and severity of GLD symptoms than observed in the source plants. However, after grafting, many plants did not test positive or showed the first symptoms before 2–3 years, which is late for an indicator and later than observed in other studies [11,13,30]; the delay in appearance of symptoms may have occurred because the plants were 3 years old when grafted, but some plants did test positive for GLRaV-3 the year after grafting. We recorded a very wide range of symptoms in Pinot noir on the four dates during the 10 years: the mean values of disease incidence at harvest (2013–2022) ranged from 0 to 88% and for disease severity, from 0.1 to 4. While symptoms were evident after veraison in most groups of plants, some groups of infected plants exhibited symptoms as soon as mid-June, and other groups of plants did not show clear symptoms either before or after veraison. This response was generally observed in the same plants every year. In summary, the viruses themselves were the main factor involved in symptom expression, quite unaffected by other factors. Something similar was recently reported by [11] for two isolates, one previously known to be mild and other strong. Although it is known that some GLRaVs are often asymptomatic [18], here GLRaV-3 was in all infected plants, in some cases with GLRaV-1 or with GLRaV-1 and 2; but in view of the massive sequencing results that are appearing, other viruses or viroids could be present—but asymptomatic—and the virome as a whole could be responsible for the plant response [4].

In [13], it was found that the visual symptom identification (VSI) is 100% effective for some GLRaV but not for others, with greater variations in the 3 years than we observed in 10 years. Although we also expected that symptoms would increase over time, we only observed a consistent increase in symptoms in July; the symptoms in infected plants in August and September tended to be lower in the latter years of the study. Although the disease incidence and severity did not increase, damage increased to the extent that a number of plants did not produce grapes in the last two years and the mean loss of infected plants was higher than 60% in the last three years of the study.

The graphs for the different indices assessing symptoms show that many of the GLRaV-3-infected plants either did not have any symptoms or displayed only very mild symptoms. If the infection remained invisible in the field in an indicator, this would surely happen in many red cultivars and in most white ones; there could be a risk in thinking that it will not be a major problem because late or absent symptoms meant an absence of economic losses (Figure 12 and Figure 13). However, these plants will still be sources of GLRaV-3 to be transmitted by mealybugs that do not require heavy infestations for the virus to spread rapidly [31] and such transmission could “create” plants with more aggressive combinations of viruses. Making decisions about roguing infected plants can be based on direct observation of GLD symptoms or tele-detection of symptoms [32] at least in red cultivars [13]. However, some infected plants could remain undetected, making some proposed roguing strategies ineffective [29].

The GLRaV-3-infected plants grafted in this study were obtained from several vineyards according to the symptoms and damage caused at origin; the vineyards were heterogenous (no clonal material available), with different cultivars, different locations, different environmental conditions and age. The molecular characters of GLRaV-3 were not associated with the phenotypical variation [15]; now we know that some of the GLRaV-3 infected source plants are in mixed infections with GLRaV-1 and/or GLRaV-2 and that could account for some of the stronger symptoms and damages in some isolates, but not always, as the preliminary data before 2017 showed [22]. However, as [4] suggested, all types of combinations of viruses and viroids or even other systemic pathogens may cause differences in GLD symptoms. If the influence of the known large genetic variation among GLRaV-3 isolates on the foliar symptoms from different grapevine cultivars remains undescribed, especially in cool-climate growing environments [13], now HTS reveals that the presence of only one GLRaV is rare and we must also consider the multiple possible combinations with other GLRaV, which in turn will also display specific genetic variations and will modulate GLD symptoms [30]. If one disease can cause such differences in clonal plant material, in more heterogeneous vineyards, growers will have difficulty in detecting and identifying infected plants that may or may not decline over time.

In a review paper published in 2013, Ref. [1] pointed out that much research had focused on the viruses, but limited attention had been given to the disease itself and the factors affecting disease development, which may vary in relation to the different viruses and also different climates and growing conditions. However, several recently published studies have considered different aspects of the GLD together with the associated viruses [4,11,13,31].

The 10-year recording of symptomatology using the different indices that we report here provided good predictors of yield losses, a decrease in sugar content, lower vegetative development and even full decline in the case of the more aggressive combinations. Sequencing of the 10 isolates (soon to be completed) will clarify whether other specific viruses, viroids or other pathogens are associated with some of the different manifestations of symptoms of GLD. Plants infected with some GLRaV-3 isolates that always had mild symptoms or remained asymptomatic 10 years after grafting did not cause damages but remained a source of infection for GLRaV vectors. It would be worth studying these isolates in depth in case they could be used in “cross protection”.

## 4. Materials and Methods

### 4.1. Location and Environmental Conditions

The experimental vineyard is located in Portomarín (Lugo, Spain, N 42.812751/−7.609543 W). The climate station (Meteogalicia, Lu-10108) is located nearby (1.6 km, same altitude 447 m, 42.813/−7.620 W); data on temperatures (Tmax, Tmin and Tavg) and rainfall (R, L/m^2^) for the decade 2013–2022 were downloaded and processed to determine any possible influence on precocity and severity of leafroll symptoms. Two indices used in viticulture to qualify the environmental conditions during the season were calculated:

$$Winkler\;index\;\left(WI\right)=\overset{31/10}{\underset{1/4}{\sum }}\left(\mathrm{Tavg}-10\right)$$

$$Huglin\;index\;\left(HI\right)=\overset{\frac{30}{9}}{\underset{\frac{1}{4}}{\sum }}\left[\left(\mathrm{Tavg}-10\right)+\left(\mathrm{Tmax}-10\right)\right]/2*k$$

*k* = correction coefficient for the mean daylight period at the studied latitude (40–50°).

### 4.2. Leafroll Virus Isolates

Beginning in 2012, 10 of the GLRaV-3 isolates characterized by [15] with GLRaV-3 in groups I and II were grafted (chip budding) onto certified Pinot noir plants (clone 115) with Gravesac (clone 264) as rootstock, planted in 2009. Three blocks of three plants of each of the ten GLRaV isolates were randomly assigned to two terraces. Six plants grafted with buds from two leafroll free plants (Al·120A, Al·D2) together with six non-grafted Pinot noir plants on both terraces were the healthy controls. Preliminary information about precocity and severity of symptoms of the mother plants in their original location was available.

### 4.3. Virus Detection

GLRaV-3 had previously been detected by ELISA-DAS and confirmed by RT-PCR in most mother plants in 2006–2009 [7,15]; GLRaV-1 and 2, were later detected in some of the mother plants and confirmed by RT-PCR [7]. None of the plants tested positive for GFLV, ArMV, GFkV; GPGV was also tested in 2019; no symptoms of those or other diseases caused by systemic pathogens or wood diseases were observed.

### 4.4. Leafroll Disease Symptoms

The GLRaVs were detected on average 1.5–3 years after grafting; GLD symptoms usually appeared in the same summer when the virus was detected. All plants were examined to detect leafroll symptoms every summer. In the early years of the study (2013, 2014 and 2015) symptoms were recorded directly in the field in mid-June, July, August and September. After 2016, the observations were recorded by taking digital photos, monthly until 2022. All the pictures were taken early in the morning before the fog rose. To evaluate the best way to record the visual identification of GLD symptoms, the following data were logged:

Disease Incidence (I): percentage of canopy surface with leaves showing “red leafroll”, independently of the severity of symptoms.

Disease Severity (S). The maximum intensity of symptoms was determined at the leaf level, without taking into consideration the spread of the symptoms in the canopy. Severity was categorized in five frequency classes: 0, no leafroll symptoms; 1, weak interveinal reddening only in old leaves; 2, interveinal leaf reddening but not intense or only in the oldest leaves; 3, typical and unmistakable symptoms, leaf rolling besides interveinal leaf reddening; 4, intense symptoms with general reddening, leaf rolling and leaves with brittle texture. In case of occasional reddening symptoms due to *Empoasca vitis* or other causes, the trained observers had to avoid recording them as GLD. The raw data were used for the statistical analysis, but a disease severity index was calculated for each date with the Townsend–Heuberger formula [33]:

$$\mathrm{DSI}\;\left(\%\right)=100*\overset{4}{\underset{0}{\sum }}\frac{\left(class\;frequency*\;score\;of\;rating\;class\right)}{total\;number\;of\;plants*disease\;index\;}$$

For each plant and date, a general index which considers both the severity and percentage of the canopy affected was calculated: IS = (I*S)/100.

Two Indices of early appearance of symptoms were calculated for 2014–2022:

Sy < V. It is a simple index with the mean number of positive plants with any leafroll symptoms before veraison, i.e., during the observations in mid-July, because for this zone and cultivar, veraison starts in August.

EI. An “earliness index” (EI) measuring the earliness of symptom outbreak was calculated for each plant and observation date in any year, using a formula that gives a decreasing factor to the date of observation of symptoms (for 4 June; for 3 July; for 2 August and for 1 September); it was applied to the Severity of symptoms for any date, i.e.:

EI=(SJun∗4+SJul∗3+SAug∗2+SSep∗1)/4

### 4.5. Harvest and Quality of Musts

At harvest (2014, 2015, 2016, 2018, 2020, 2021, 2022), the number of clusters (NoC) and weight of all grapes (Y) were recorded for each plant. Must was obtained from 50 grapes randomly picked at harvest from both sides of the plants and analyzed for sugar content (°Brix) with a handheld refractometer and total acidity (TA, mg/L tartaric acid) with an autoanalyzer (Atago PAL-Easy ACID2, Japan). The weight of the prunning wood (PW) was determined in January 2019, 2022, and 2023.

In 2013 and 2014, only Y and NoC were recorded. In 2017, the mentioned spring frost on 28 and 29 April destroyed the canopy, and therefore there are no harvest data for that year. In 2019, an infection by downy mildew late in the season affected clusters irregularly and the data are therefore not representative.

### 4.6. Statistical Analysis

For the statistical analysis, for each year, we used only data from plants that had already tested positive for GLRaV-3 at harvest the previous year; once all were positive, we used the 90 infected (for symptom indices) or all 108 plants, including the negative controls (for yield and must data).

Scatterplots of the indices of symptom expression (I, Se, I*S, DSI or earliness (Sy < V and EI) were constructed, and Pearson’s coefficients were used to assess the correlations between variables related to symptoms, environmental conditions, years infected and yield; regression analysis was also conducted for some comparisons, especially those between any of the indices of symptoms and data on yield/plant and quality of the must from each plant.

The effect of the isolates in the infected groups of plants was analyzed following a general linear model (GLM) and univariate analysis of variance (one way ANOVA) with the symptom indices and harvest data as dependant variables, the isolates as independent variables, and the repetitions of three plants as random-factor analysis.

All statistical analyses were performed using IBM—SPSS Statistics 27 sofware.

## Figures and Tables

**Table 1:** Main meteorological data for the growing period of the grapevine (April to September) between 2013 and 2022. Data downloaded from Meteogalicia station at Portomarín (Lugo, Spain). R, total rainfall; Tmx, Tm and Tmn, maximum, mean and minimum temperatures respectively, between April and September; A: April, S: September. Year classification according to the Winkler index (WI) and the Huglin heliothermal index (HI).

Year	R (L/m^2^) (A-S)	Tmx (°C)	Tm (°C)	Tmn (°C)	Min abs (A-S) °C	Date	WI °C	Class W	HI °C	Class HI
2013	274.6	23.2	15.3	9.0	−1	6 April 2013	1172	1b	1830	HI−1
2014	256.6	23.5	15.8	9.8	1.7		1227	1b	1941	HI−1
2015	218.5	24.5	16.3	9.7	2.9		1249	1b	2019	HI−1
2016	338.8	20.8	15.2	10.6	0.6		1106	1a	1596	HI−2
2017	216.4	25.1	16.7	10.1	−2.8	28&29 April 2017	1376	1b	2118	HI+1
2018	286.8	20.9	15.6	11.4	2		1175	1b	1636	HI−2
2019	279.4	23.4	15.8	9.7	0.5		1211	1b	1879	HI−1
2020	282.7	24.5	16.9	11.1	1.6		1326	1b	2071	HI−1
2021	261.3	23.0	15.5	9.7	−1.4	12&17 April 2021	1150	1b	1809	HI−1
2022	213	24.6	16.7	10.7	−4.3	5 April 2022	1451	1b	2115	HI+1
Mean	262.8	22.8	15.7	10.1			1244.3	1b	1901	HI−1
±sd	39.2	1.4	0.5	0.6			108.64		186.9	

**Table 2:** Mean, maximum and minimum temperatures in June, July, August, and September. Mean values for 2013 to 2022.

	Tm °C				Tmx °C				Tmn °C				R (mm)			
Year	Jn	Jl	Au	Sp	Jn	Jl	Au	Sp	Jn	Jl	Au	Sp	Jn	Jl	Au	Sp
2013	15.1	10.2	14.9	20.6	15.9	17.0	22.8	29.9	8.4	14.0	11.9	11.1	37.8	10.8	11.0	50.6
2014	15.7	12.6	16.5	18.5	23.2	20.2	24.7	26.2	9.9	12.1	11.3	12.4	25.6	29.0	27.6	57.2
2015	15.8	14.2	17.9	19.9	18.9	21.9	27.2	28.7	10.7	12.4	12.4	9.6	3.4	13.8	27.6	56.8
2016	15.0	12.5	15.6	18.4	13.3	17.3	20.7	24.3	10.8	13.8	13.2	12.1	39.2	2.6	12.6	49.0
2017	16.3	15.4	18.4	19.4	21.3	23.1	26.3	27.7	12.3	13.0	12.3	9.6	42.0	12.2	34.6	15.2
2018	15.3	12.8	15.9	17.9	14.5	17.8	20.4	22.4	12.1	13.9	13.7	13.5	84.4	20.0	8.4	10.2
2019	15.4	13.4	15.3	19.2	17.8	20.4	22.4	24.9	9.6	12.9	13.3	11.1	44.2	47.2	18.8	18.0
2020	16.2	16.3	16.3	20.2	16.6	21.4	22.2	27.6	6.3	20.2	19.0	17.2	19.6	0.5	67.6	59.6
2021	15.2	12.6	16.0	18.2	17.8	24.2	23.4	29.1	10.7	12.2	12.5	11.4	57.1	6.3	14.5	43.8
2022	16.6	15.5	16.7	20.8	17.3	23.6	23.1	29.9	11.6	13.6	14.6	11.2	98.1	6.2	10.3	33.7
Avg	15.7	13.5	16.4	19.3	17.7	20.7	23.3	27.1	10.2	13.8	13.4	11.9	45.1	14.9	23.3	39.4
sd	0.6	1.8	1.09	1.05	3	2.6	2.2	2.6	1.81	2.34	2.17	2.19	28.6	14.1	17.9	18.8

**Table 3:** Correlations (Pearson’s r) between symptom indices and yield (AvgY) or must quality parameters (AvgBrix and AvgTA) and mean KgxBrix. Mean values for 2013–2022 for all plants.

	AvgY	AvgBrix	AvgKgxBrix	AvgTA
Sy < V	−0.70 **	−0.57 **	−0.72 **	0.14 ns
EI	−0.75 **	−0.64 **	−0.77 **	0.17 ns
IJl	−0.67 **	−0.45 **	−0.66 **	0.10 ns
IAu	−0.65 **	−0.69 **	−0.68 **	0.24 *
ISp	−0.66 **	−0.70 **	−0.70 **	0.33 **
SJl	−0.72 **	−0.55 **	−0.73 **	0.17 ns
SAu	−0.73 **	−0.70 **	−0.76 **	0.23 *
SSp	−0.70 **	−0.72 **	−0.74 **	0.31 **
I*S	−0.63 **	−0.68 **	−0.65 **	0.35 *

ns, non significant; *, ** significant with *p* < 0.05 and *p* < 0.01 respectively.

**Table 4:** Analysis of variance for several symptom indices when the 10 virus isolates present in Pinot noir are the independent variable.

Dependent Variable	df	F	*p*<
EI	9	68.379	0.000
Sy < V	9	46.996	0.000
S July	9	53.468	0.000
S August	9	101.435	0.000
S September	9	134.718	0.000
I July	9	32.571	0.000
I August	9	119.909	0.000
I September	9	162.202	0.000

**Table 5:** Analysis of variance for yield, vegetative growth (prunning weight) and quality of must (average for 2013–2022) when the 10 virus isolates present in Pinot noir (+ control plants) are the independent variable.

Dependent Variable	df	F	*p*<
No clusters	10	25.803	0.000
Yield	10	32.621	0.000
Pruning weight 2023	10	23.247	0.000
TSS	10	43.869	0.000
Total acidity	10	3.201	0.002
Kg*Brix	10	38.539	0.000

## Data Availability

Raw data are available upon request.
